# Calcium element quantification model using a portable X-ray fluorescence unit

**DOI:** 10.1016/j.mex.2023.102287

**Published:** 2023-07-12

**Authors:** Claudio H. González-Rojas, Cristián Castro-Rodriguez, Sebastián Gutiérrez-Vivanco, Esteban Vargas-Vera

**Affiliations:** a Department of Chemistry, Faculty of Sciences, Universidad de Tarapacá, Velásquez Av. 1775, XV Region, Box 7-D, Arica, Chile; b Archaeometric Analysis and Research Laboratory (LAIA), High Research Institute, Universidad de Tarapacá, Box 7-D, Arica, Chile

**Keywords:** Calcium, Portable, X-ray fluorescence, Quantitative, Determination, Calcium quantitative analyses

## Abstract

A methodology is proposed to achieve an effective quantification of the element Calcium in soil samples, using a portable X-ray Fluorescence (pXRF) equipment. The protocol began by preparing two ideal matrices using an increasing mass, measured on an analytical balance, of Calcium and Sodium Nitrate as binder. It is the Gravimetric technique. In both samples the fluorescent emission line of the element Calcium was calibrated using an standard reference material 1400 boneash.

The validation of ideal samples of the element Calcium using the XRF technique with respect to AAS as an experimental standard, using linear correlation methods, to estimate from statistical inference the real concentration of the analyte, allowed us to appreciate that both approaches remain above the standard curve provided by AAS, with a value of 1.8 and 1.68, respectively. Which means that the tendency to evaluate Calcium samples by XRF are overestimated with respect to the curve provided by AAS. Since the last correlation coefficient is very close to 1.0 and its statistical inference equation is precisely known, the authors of this work propose to apply quantification protocol to real sediment samples.

• A methodology is proposed to achieve an effective quantification of the element Calcium in soil samples, using a portable X-ray Fluorescence (pXRF) equipment
• The validation of ideal samples of the element Calcium using the XRF technique with respect to AAS as an experimental standard, using linear correlation methods
• This work pretend to resolve another weakness by the portable system and is that the number of accounts provided by conventional XRF are not consistent with a% w/w of analyte.

## Graphical abstract

**Figure:**
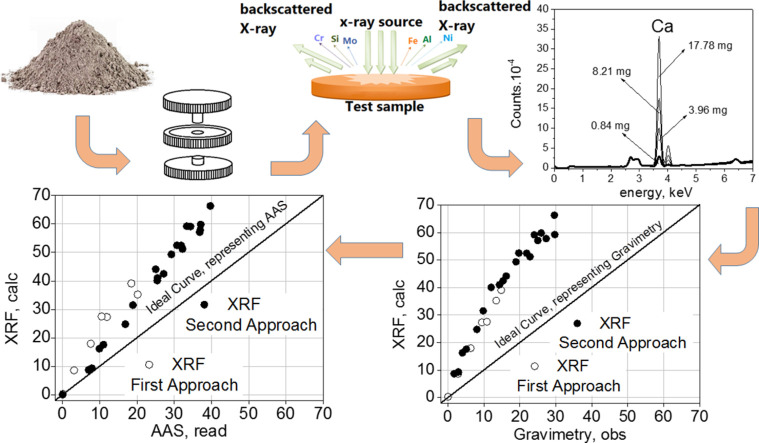

Specifications Table

**Table:** 

Subject area:	Chemistry
More specific subject area:	Quantitative analyses
Name of your method:	Calcium quantitative analyses
Name and reference of original method:	N.A.
Resource availability:	Portable X-ray fluorescence, manual pill box https://www.brukersupport.com/ProductDetail/9267, Excel or Rstudio CaCO_3_, NaNO_3,_ 1000 ppm Calcium titrisol and Calcium interferent suppressors analytical balance, alcohol denaturalized, 98%, cleaning towel,

## Method details

### XRF

X-ray fluorescence, or XRF, is the emission of secondary (or fluorescent) X-rays produced by material that has been excited with high-energy X-rays or gamma rays[1]. This phenomenon is widely used for elemental and chemical analysis, particularly in studying metals, glass, ceramics, building materials, geochemistry, forensic science, and archaeology[[2], [3], [4]] and [5].

**Fig. 1:**
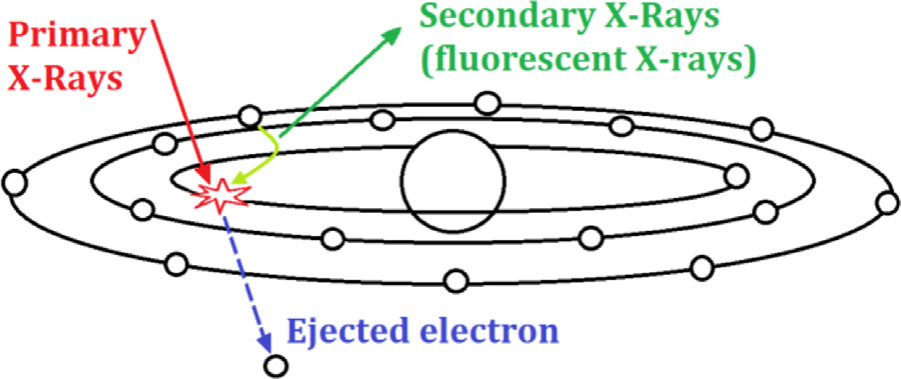
X-ray fluorescence, property of the author.

Both X-rays and gamma rays can be powerful enough to detach tightly bound electrons in the inner orbitals of the atom (Fig. 1). Such electron removal leaves the atom's electron structure unstable, and electrons in higher orbitals decay into the vacancy generated by emitting a photon. The orbitals distribution and the number of electrons in those orbitals are periodic properties of each element, as is the energy difference of the orbitals participating in this transition. So the energy of this fluorescent photon is characteristic of the atoms that make up the material.

The determination of elements in situ in the soil matrix aims to achieve an increasingly practical diagnosis of contamination by heavy metals in natural or remote sites.

### Sample preparation

#### X-ray fluorescence spectrophotometry

##### First test with ideal matrices

**Fig. 2:**
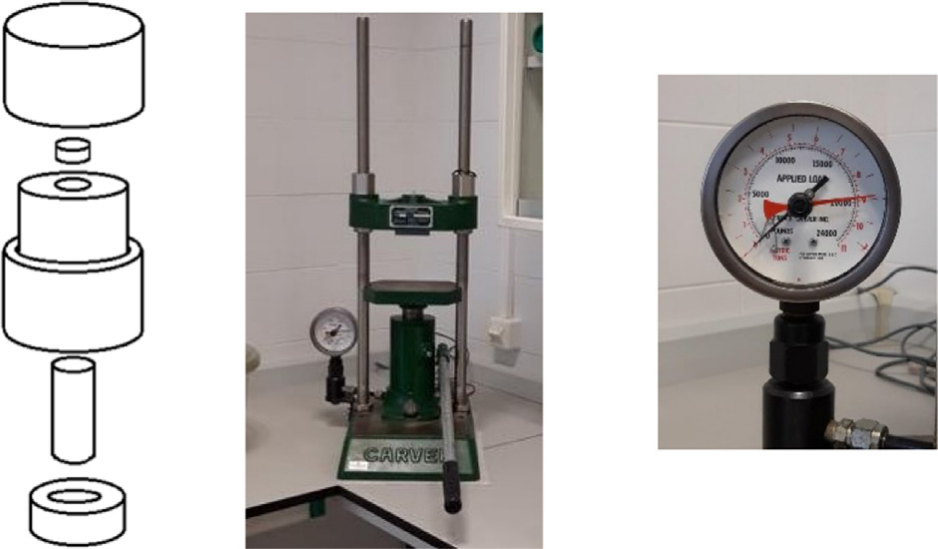
Mold for pill press, reproduction of the original, turnery workshops, Universidad de Tarapacá, property of the author, and CARVER lab Model C Manual Press for IR, First test with glucose matrix.

The most suitable XRF methodology for measure Ca concentration requires a solid matrix. The matrix has to satisfy several requirements; one of them is to not interfere with Calcium and other elements present in the soil under study. Glucose was proposed as the first matrix test. Glucose does not emit XRF, as it is suitable from second-period elements forward; glucose can be agglomerated as a pressed pellet. Therefore, glucose is a suitable matrix. Following this, samples of increasing concentration of Calcium were prepared in pellets. CaCO_3_ was used as the analyte test standard. The tableting box used and Manual Press is presented in Fig. 2.

##### Second test with ideal matrices

The samples were prepared from CaCO_3_ Fluka. The matrix was NaNO_3_ Merck. The contrast ion CO_3_^2−^ was chosen in the case of Ca and NaNO_3_ since they do not emit X-ray fluorescence. NaNO_3_ can also be agglomerated as a pressed pellet and therefore appears as a suitable matrix. All Ca reagents and NaNO_3_ were dried in an oven previously for 12 hr before sample preparation.

**Fig. 3:**
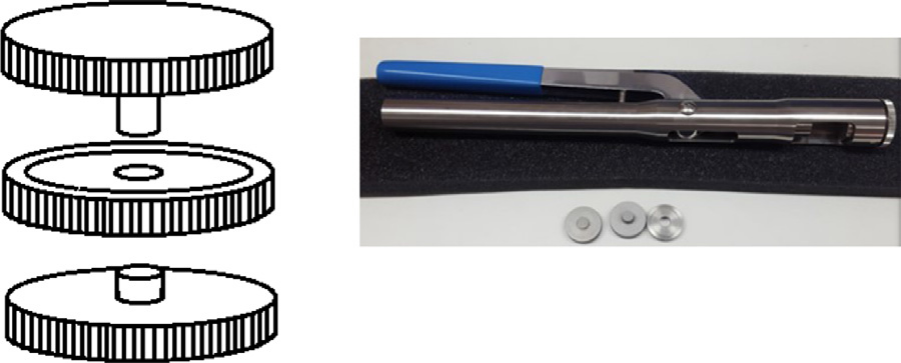
Tableting box, property of the author, and hand press used from PE instruments. Second test with NaNO_3_ matrix.

Each dehydrated sample was pulverized using an agate mortar and further processed using pressed tablets. The PE 01,860,436 QuickPress w/7 mm die & Pellet Extractor is shown in Fig. 3 and other accessories provided by Perkin Elmer General Purpose FT-IR Accessory kit, L136–5312.

#### Portable X-ray fluorescence system (pXRF)

**Fig. 4:**
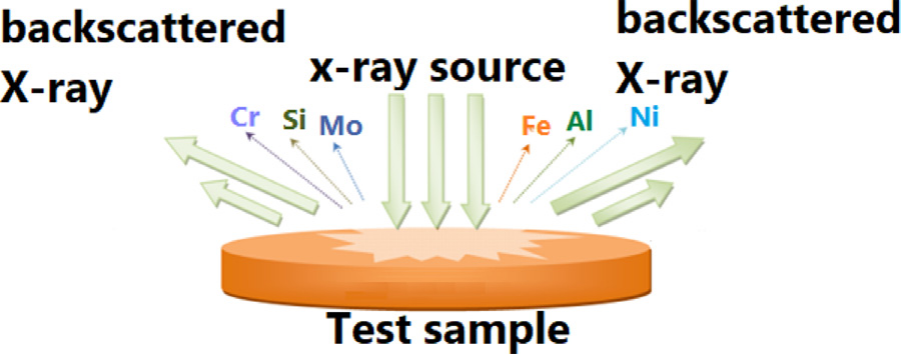
Tablet formation scheme, property of the author.

X-ray analysis was performed in a Bruker Trace III-SD device. The potential used was 30 kV and a current of 37.8 μA, with an exposure of 180 s for each sample. Below a scheme (Fig. 4)

#### Calcium standards for X-ray fluorescence spectroscopy

The X-ray fluorescence spectrum of a standard material was recorded; the standard material chosen was NIST SRM 1400 Bone Ash containing 38.18% (w/w) of the Calcium element. The area under the curve integrated into the Calcium line located at 3.67 keV reaches a dimensionless value of 6,834,285; this value was considered to compare with the masses interpolated from the NaNO_3_ matrix assay.

Once measured by XRF, the matrices of the first and second assays were processed, and the Ca content was compared by AAS.

#### Atomic absorption spectrophotometry

A Varian RMX 300 AA200 with a flame emitter and Calcium lamp at a wavelength of 256 nm was used. The beam was coaligned, and the flame used was acetylene and air as oxidant. The length of the optical path was 10 cm, and the ratio of acetylene to air was 1:3 by volume. The burner was used in a 90 °C configuration, and the blank was 10% HCl.

#### Sample digestion for use in atomic absorption spectroscopy

The pellets were processed in HCl/HNO_3_ mixture using EPA 3050B Method [6], with boiling beads, and then brought to dryness, where 100 mL of 10% HCl was added, heated to boiling, and then allowed to cool. Once cooled, the samples are filtered and received in a 250 mL volumetric flask. Carefully the filter paper was washed with small volumes of 10% HCl until the determination was completed.

#### Calcium standards for atomic absorption spectroscopy

Calcium standards were used under two different conditions: without Calcium interference suppressors and with suppressors. Calcium titrisol diluted in a battery of solutions was used.

## Results and discussion

### X-ray fluorescence spectroscopy (XRF) analysis

#### First matrix test

**Fig. 5:**
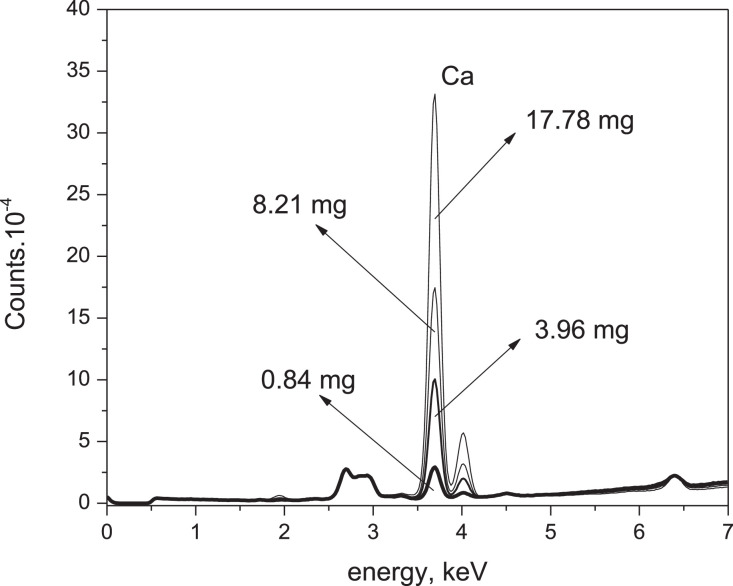
CaCO_3_ emission lines in solid solution at four different compositions.

**Fig. 6:**
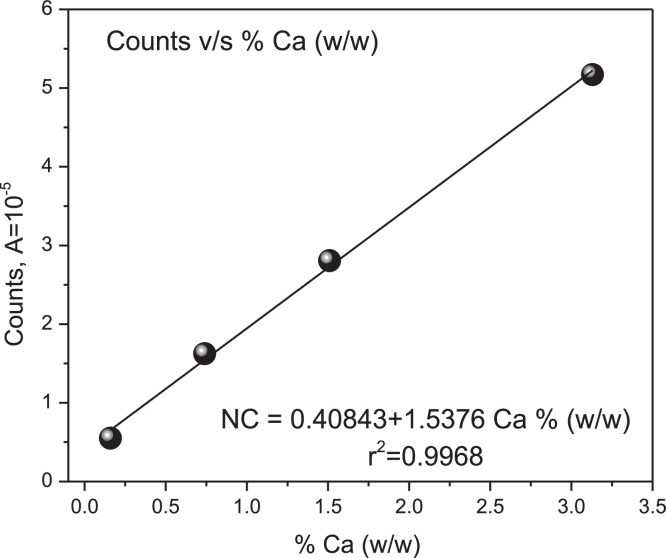
Linear Regression Curve. Number of counts XRF v/s % Calcium mass.

For the second objective, an appropriate observable seems to be the number of beads. This parameter represents the amount of X-radiation fluorescence photons; the radiation should be proportional to the Ca element population. Table 1 summarizes the Ca, glucose, and the number of beads concentrations. Fig. 5 shows the CaCO_3_ emission line in four different compositions, and Fig. 6 shows the correlation curve between this parameter and the concentration.

**Table 1:** Formation of ideal Ca analyte pellets in glucose matrix according to analyte mass, number of beads and area under the emission line according to XRF.

	CaCO_3_, mg	Glucose mass mg	Total mass mg	Ca, mg	% (w/w) mg Ca/mg Total mass	Max NC Number counts	Area dimensionless
1	2.1	521.8	523.9	0.84	0.16	29,152	5505
2	9.9	524.6	534.5	3.96	0.74	100,413	16,235
3	20.5	524.3	544.8	8.21	1.51	174,588	28,068
4	44.5	521.8	566.3	17.78	3.13	331,377	51,712

PM. CaCO3 : 100.088 g/mol : Ca : 40.08 g/mol.

The beads are read on the energy line corresponding to Ca:3.70 keV.

Each tablet was subjected to XRF. At the Calcium emission line position, the number of beads was measured and ordered according to the mass percentage of Calcium relative to the mass of glucose, (%w/w). The first test of ideal Calcium tablets in the ideal glucose matrix and a table showing the correlation between the two variables are presented below.

Fig. 5 shows an increase in the number of beads proportionally to the mass of Calcium. Therefore, the number of beads appears as an observable suitable to correlate with the solid phase concentration of the Calcium element. Fig. 6 shows the simple linear regression between the number of beads read on the Calcium emission line. Its Pearson correlation coefficient adjusted according to a linear fit reaches a value of R^2^ = 0.9968. This correlation is strong and positive.

Glucose, meanwhile, behaves as a suitable matrix in the solid phase since no interference is observed in the number of beads read as a function of the calcium mass. The following assay made use of another NaNO_3_ matrix and is examined below.

### Second test of ideal matrices

#### First approach with NaNO_3_ matrix

The second test was carried out with the NaNO_3_ matrix, with which two approximations and the search for ideal supports for XRF were performed. The results are presented below.

Table 2 is presented below. It contains the ideal NaNO_3_ matrix pellets and other parameters derived from the XRF emission.

**Table 2:** Formation of ideal pellets of Calcium analyte in NaNO_3_ matrix, according to mass of analyte, number of counts and area under the emission line according to XRF (first approximation).

	CaCO_3_, mg	NaNO_3_ Mass mg	Total Mass mg	Ca, mg	% (w/w) obs	Max NC Number counts	Area dimensionless
1	0	62.9	62.9	0	0	9620	51,456
2	4.8	64.0	68.8	1.9	2.79	211,646	1,546,256
3	12.3	65.4	77.7	4.9	6.33	434,475	3,214,503
4	19.6	63.8	83.4	7.8	9.40	658,101	4,885,137
5	23.7	63.9	87.6	9.4	10.82	664,614	4,930,072
6	31.5	62.4	93.9	12.6	13.42	847,321	6,307,775
7	36.9	63.0	99.9	14.7	14.77	937,069	6,990,804

PM. CaCO_3_ : 100.088 g/mol: Ca : 40.08 g/mol.

^1^ NaNO_3_ pure Area: by mean of numerical integration.

##### Calcium observed by gravimetry

**Fig. 7:**
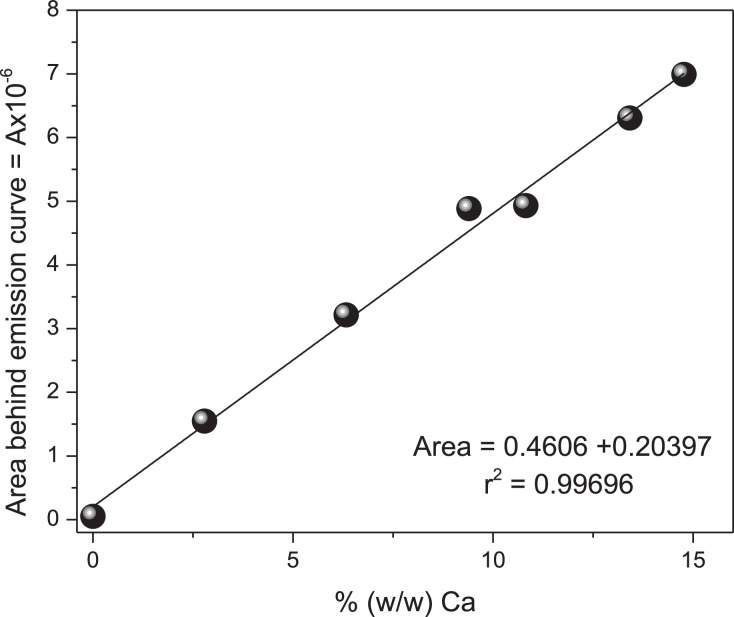
Calculated area for CaCO_3_ emission lines in NaNO_3_ matrix at six different compositions (first approximation). NaNO_3_ matrix.

Based on this Table, we correlated Calcium% observed versus the integrated area under the Calcium XRF emission line. The area could be a better variable than the number of beads as it contains more information than the maximum observed. The area is represented by a population of energy data around the central maximum, as opposed to the maximum reading, NC, represented by a single line. Within this context, the area appears to be a more meaningful indicator of Calcium concentration. Fig. 7 presents the correlation between the area under the observed Calcium emission line and the observed Calcium mass percentage. According to Pearson, its linear correlation coefficient is 0.9970 indicating a strong and positive correlation.

##### Calcium calculated by XFR

**Fig. 8:**
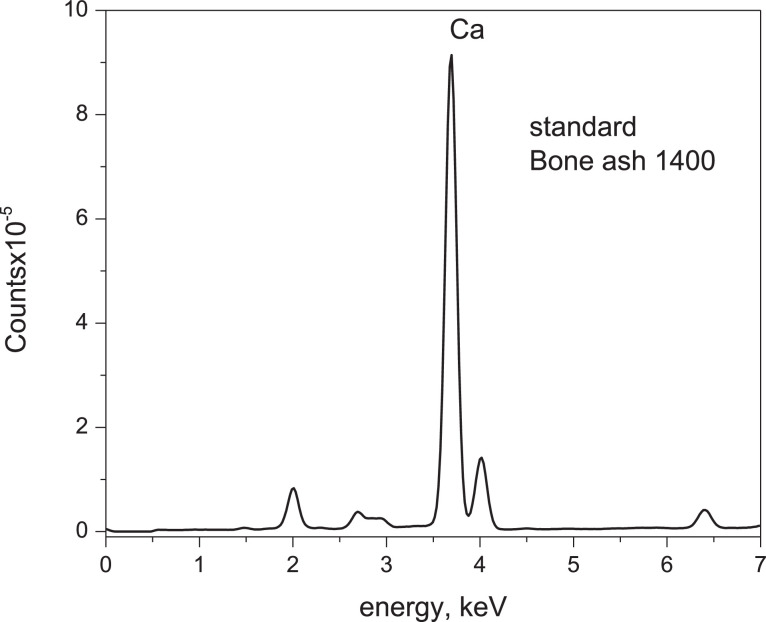
Standard Ca emission lines Bone ash 1400.

Calcium% (w/w), calculated Ca, were then estimated and compared with the same mass of Calcium, observed Ca, to determine the proportionality factor between observed Calcium and calculated Calcium. Previously, the spectrum published in standard literature was registered, Bone Ash 1400, to evaluate the area under the curve for the XRF emission line of the Calcium element, see Fig. 8.

The area data of the Ca element from Table 3 was then taken, and each value of the estimated A_calc_ area was converted to the ratio indicated below for the decimal percentage determination of the Calcium analyte compared to the Bone Ash 1400 standard; therefore, the value of x is in percent per one:

(1) $$\begin{array}{c} \frac{6,834,285}{0.3818}\;=\frac{A_{\mathrm{calc}}}{x} \end{array}$$

**Table 3:** Comparison between the experimentally observed masses of the element Calcium by gravimetry, the masses calculated by XFR, pattern area,% (w/w) of Calcium calc, mass in g of this element, and the proportionality factor between both determinations. Matrix in use: NaNO_3_.

	Pellet Mass mg	Calcium Mass mg	% (w/w) Calcio obs	Area_calc_ dimensionless	% (w/w) Calcium calc	Calcio calc Mass	Proportional Factor of obs/calc
1	62.9	0	0.0	51,456	0.2875	0.1808	0.0
2	68.8	1.9	2.8	1,546,256	8.6382	5.9431	3.09
3	77.7	4.9	6.3	3,214,503	17.9579	13.9533	2.85
4	83.4	7.8	9.4	4,885,137	27.2910	22.7607	2.90
5	87.6	9.4	10.8	4,930,072	27.5420	24.1268	2.55
6	93.9	12.6	13.4	6,307,775	35.2386	33.0891	2.63
7	99.9	14.7	14.8	6,990,804	39.0544	39.0153	2.64

M: Pellet mass (100% mass)							2.7 ± 0.2

The percentage mass value of the element Calcium %(w/w) from this ratio was calculated as follows:

(2) Ca=x·100%,%(w/w)

The next step was to compare the gravimetric composition of the Ca analyte in % Ca (w/w), hereinafter Calcium observed (obs), to the results obtained for the same pellet using XRF, hereinafter Calcium calculated (calc). These results are presented in Table 3.

**Fig. 9:**
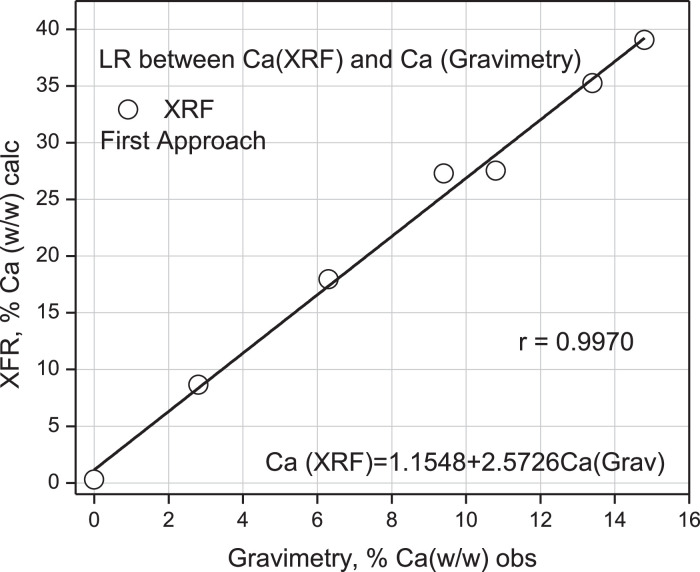
Linear regression (LR) of Calcium element % (w/w) calc from XRF spectroscopy versus Ca % (w/w) obs from Gravimetry. First approach. Matrix used: NaNO_3_.

As can be seen in Table 3, the calculated percentage % (w/w) of calcium compared to the observed one is overestimated by a factor of 2.7 on average, with an estimation error of 0.2%. Nevertheless, a simple linear regression between both sets of values allowed obtaining a Pearson correlation coefficient of 0.9970, which indicates a positive and strong correlation, as shown in Fig. 9. A correlation of this type agrees with the correlation found in the literature for Calcium compounds [7].

The second approach will discuss the proportionality factor between the two determinations.

This first approximation allows concluding that a predicted estimate of the Calcium element value in an actual sample, equivalent to that observed by Gravimetry, should correspond to the predicted value, following the linear regression found, see Fig. 7, in the form:

$$Ca\left(real\right)=\frac{\left(Ca(calc)-1.1548\right)}{2.5726}$$

It is expected that this result will be generalized once the second approximation is achieved.

Both ideal matrices, glucose and NaNO_3_ were up to this point suitable for mass determinations of the analyte calcium. However, NaNO_3_ was preferred to glucose for the discussion that follows. The reasons will be addressed again when the analysis of assays in both matrices using AAS has been completed.

#### Second approximation with ideal NaNO_3_

##### Calcium observed by gravimetry

The second approach involved experimenting with a larger number of pellets to increase the statistical significance of this study while conserving the NaNO_3_ matrix. The results are presented in Table 4.

**Table 4:** Formation of ideal Ca analyte pellets in NaNO_3_ matrix, according to CaCO_3_ mass, NaNO_3_ mass, total mass (pellet), and the mass of the analyte obs. and % (w/w), second approach.

	CaCO_3_ mg	NaNO_3_ mg	Total Mass mg	Ca obs mg	Ca obs % (w/w)
1	0.0	0.0	0.0	0.0	0.0
2	3.0	66.9	69.9	1.2	1.7
3	5.1	65.1	70.2	2.0	2.9
4	7.0	63.6	70.6	2.8	4.0
5	8.9	61.1	70.0	3.6	5.1
6	14.1	56.3	70.4	5.6	8.0
7	17.1	53.0	70.1	6.8	9.8
8	21.1	49.1	70.2	8.4	12.0
9	25.1	45.2	70.3	10.1	14.3
10	27.0	43.7	70.7	10.8	15.3
11	29.3	43.1	72.4	11.7	16.2
12	33.0	36.9	69.9	13.2	18.9
13	34.5	35.5	70.0	13.8	19.7
14	37.9	31.3	69.2	15.2	21.9
15	39.5	29.9	69.4	15.8	22.8
16	42.2	28.1	70.3	16.9	24.0
17	43.8	26.3	70.1	17.5	25.0
18	46.3	25.4	71.7	18.5	25.9
19	47.9	22.3	70.2	19.2	27.3
20	59.9	20.9	80.8	24.0	29.7
21	51.6	18.2	69.8	20.7	29.6

PM. CaCO_3_ : 100.088 g/mol: Ca : 40.08 g/mol.

##### Calcium calculated by XRF spectroscopy

**Fig. 10:**
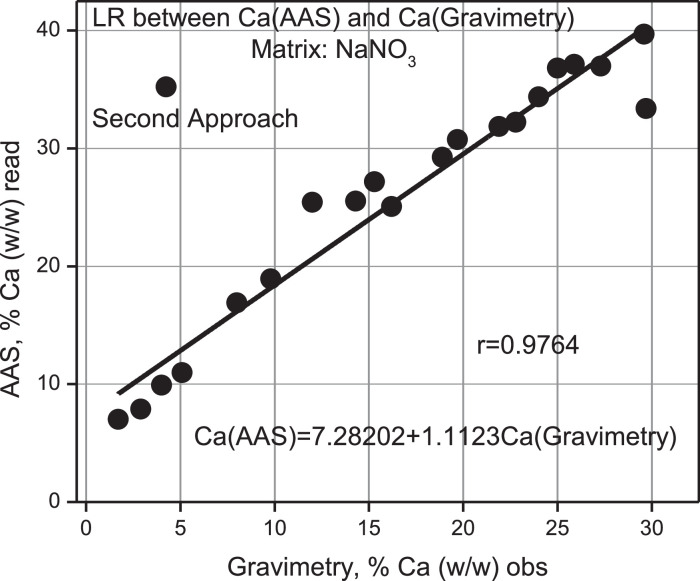
Linear regression (LR) between Ca (AAS)% (w/w) read versus Ca (Gravimetry)% (w/w) obs. Second approach.

Again the % Calcium versus the integrated area under the XRF emission line of Calcium was correlated, continuing the experience in 1.2.1.2. Eqs. (1) and 2 and the evaluated areas of the Bone Ash 1400 standard were used for analysis; the area under the Calcium emission line was measured according to each pellet and compared to the same area of the Calcium element standard, see Fig. 10.

Each value of the estimated A_calc_ area was calculated in the proportion specified in Eqs. (1) and 2 for the decimal percentage determination of the Calcium analyte. The achieved results are shown in Table 5.

**Table 5:** Pellet mass, Calcium mass,%(w/w), Area, Calcium calc (%w/w), calcium mass and proportionality factor.

	Pellet Mass mg	Calcium Mass obs mg	% (w/w) mg/mg Calcium obs	Area_calc_	% (w/w) mg/mg Calcium calc	Calcium mass calc mg	Proportionality Factor obs/calc
1	0	0	0	–	0	0	–
2	69.9	1.2	1.7	1,550,874	8.66	6.1	5.04
3	70.2	2.0	2.9	1,660,969	9.28	6.5	3.19
4	70.6	2.8	4.0	2,905,164	16.23	11.5	4.09
5	70.0	3.6	5.1	3,152,884	17.61	12.3	3.46
6	70.4	5.6	8.0	4,433,775	24.77	17.4	3.09
7	70.1	6.8	9.8	5,636,440	31.49	22.1	3.22
8	70.2	8.4	12.0	7,169,436	40.05	28.1	3.33
9	70.3	10.1	14.3	7,335,199	40.98	28.8	2.87
10	70.7	10.8	15.3	7,599,458	42.45	30.0	2.78
11	72.4	11.7	16.2	7,901,751	44.14	32.0	2.72
12	69.9	13.2	18.9	8,828,889	49.32	34.5	2.61
13	70.0	13.8	19.7	9,398,991	52.51	36.8	2.66
14	69.2	15.2	21.9	9,381,284	52.41	36.3	2.39
15	69.4	15.8	22.8	9,161,696	51.18	35.5	2.25
16	70.3	16.9	24.0	10,583,708	59.13	41.6	2.46
17	70.1	17.5	25.0	10,219,378	57.09	40.0	2.28
18	71.7	18.5	25.9	10,703,634	59.80	42.9	2.31
19	70.2	19.2	27.3	10,343,782	57.79	40.6	2.11
20	80.8	24.0	29.7	10,598,725	59.21	47.8	1.99
21	69.8	20.7	29.6	11,859,249	66.25	46.2	2.24

	Gravimetry	Gravimetry	Gravimetry	XRF	XRF	XRF	2.9 ± 0.5

As shown in Table 5, the percentage % (w/w) of calcium calculated compared to the observed value is overestimated by a factor of 2.9 on average with an estimation error of 0.5%. Nonetheless, a simple linear regression between both sets of values yielded a Pearson correlation coefficient of 0.9764, which indicates a strong positive correlation, see Fig. 10.

**Fig. 11:**
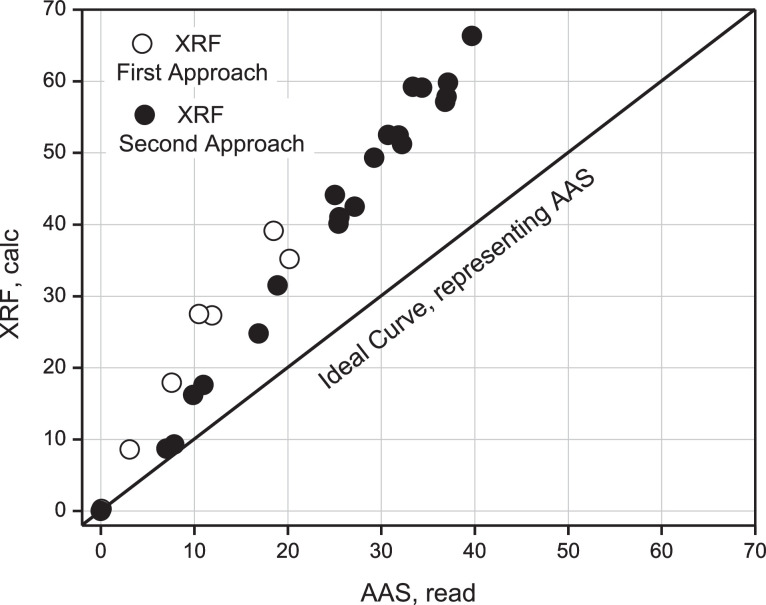
Comparison between ideal curve (AAS) representing read values, XRF calculated values from first approach and XRF from second approach.

This Table proposes to use the proportionality factor instead of the error committed by both techniques because the proportionality value is a measure of the separation between both experimental values, having better attributes than the relative error between both measurements, given that the tendency in the latter indicates that both values are increasingly separated, and therefore the error is also increasing. The separation can be seen graphically in Fig. 11; between the first and second approximation, the diagonal represents the curve provided by AAS as a measure of the read value and, therefore, the real value that should be reached with the instrumental determination using XRF.

Based on this figure, both experimental determinations are above the real or gravimetric (ideal) curve. Consequently, the tendency to evaluate actual samples is overestimated compared to the ideal curve.

Validation of ideal samples of the Calcium element using the XRF technique in comparison with the gravimetric technique as an experimental reference using linear correlation methods to estimate the actual concentration of the analyte statistically showed that both approaches, with a value of 2.7 (See Table 3) and 2.9 (See Table 5), show a tendency to overestimate the actual values. Inferences of inaccuracy regarding gravimetry can be made. However, both show good precision in determining the Calcium analyte according to the linear fit according to Pearson.

Additionally, the first approximation has a better quality linear correlation than the second and thus is more accurate. However, the second one has a larger statistical population than the first one, which means that, although less accurate, it is more representative due to the sample size examined. The following experimental protocol for actual samples could be postulated in this context and is shown below.

### Proposal for analysis of study samples

From the quantitative point of view, the protocol to estimate the mass percentage amount of the element Calcium in a test sample would consist of taking a representative sample of 30 mg, with a total mass of 50 to 60 mg of NaNO_3_ and pressing it, measuring the area under the Calcium emission line (A_calc_) and finally applying the following relation that represents an interpolation using the last simple linear regression equation reached in Fig. 11:

$$\%\text{Ca}\left(w/w\right)\;=\;\frac{\left(\frac{A_{\text{calc}}\cdot 0.3818\cdot M}{6,834,285}-9.91204\right)}{1.92191}$$

Whenever Bone Ash is used as the standard substance, M is the total mass of the pellet. This test could be extended with the assistance of other standard Calcium-containing materials, but for now, it will be left as a matter to be addressed in subsequent work. Additionally, using % (w/w) in this representation allows for measuring the Calcium element in terms of normalized mass. Therefore, two different samples having Calcium in different proportions can be better compared.

## Analysis by atomic absorption spectrophotometry

The interest in endorsing a semiquantitative technique with XRF led to comparing these measurements with a standardized technique for analyzing metals such as AAS.

Regarding the contrast between the calculated concentration of the Calcium element and the concentration observed employing the calibration curve compared to a Calcium standard using AAS, two procedures were adopted; the first one consisted of measuring the concentration of Calcium in processed samples in an HCl-HNO_3_ mixture and constructing a calibration curve for Calcium standards without Calcium interferent suppressor. Also, the second one meant the same procedure described above but adding in the solutions to be measured and interferent suppressors for the Calcium element in the calibration curve. The suppressor chosen was La_2_(NO_3_)_3_ and LiCl. This procedure is shown below.

### Analysis with/without Calcium element interferent suppressors

First, a Calcium standard was prepared from Calcium titrisol. The calibration curve of Calcium without suppressors allowed arriving at the linear fitting function presented in Table 3. Following this, this curve was used to contrast the Calcium dissolved in the pellets with ideal matrices, and the concentration deduced from this calibration curve without interfering suppressors for Calcium. The results are presented in Table 6. Table 7

**Table 6:** Calibration curve for Ca standard. Ca with and without suppressors (*).

	ppm	Abs	
1	0	0.0006	Without suppressors
2	20	0.0064	Abs = 3.3.10^–4^C + 2.9.10^–4^
3	40	0.0137	r^2^= 0.9993
4	60	0.0202	
5	80	0.0266	
6	100	0.0335	

1	0 *	0.0097	With suppressors
2	10 *	0.1075	Abs = 0.01053 C + 0.016
3	20 *	0.2476	*r* = 0.9744
4	30 *	0.3889	
5	40 *	0.4320

**Table 7:** Calcium concentration in HCl (obs) and Calcium concentration deduced from the standardization curve (read) of Ca with and without suppressors (*) in glucose matrix.

	Ab	Ca ppm obs	Ca ppm read.	% desv.
1	0.003	8.2	8.4	2.4
2	0.013	38.5	39.6	2.8
3	0.025	74.9	82.1	8.8
4	0.050	150.6	177.8	15.3

			average	7.3

1	0.1820 *	15.8	14.7	−7.5
2	0.2623 *	23.4	21.3	−9.9
3	0.3590 *	32.6	33.2	1.80

			average	5.2

The % deviations correspond to the relative difference of the read value according to the linear regression curve compared to the observed value of the analyte by gravimetry. In both cases, it can be seen that the relative difference is slightly above 5% error. The measurements with suppressors were slightly more accurate than those without suppressors. These results show that glucose is a suitable matrix for studies. However, in a global context, it evidenced some concerns. During processing, it tended to caramelize; then, there could have been retained substrate in evaluation at this stage insoluble in hydrochloric acid. Therefore, it was discarded in subsequent trials and was preferred to work with NaNO_3_ as the ideal matrix. Acid digestion is also a critical step since the substrate used comes in the form of CaCO_3_, and this compound has a solubility product. Therefore, the sample preparation must be very well executed.

#### Acid processes of Calcium tablets dissolved in ideal NaNO_3_ matrix

##### First approximation

The NaNO_3_ matrix is very soluble in an acidic environment, making it a satisfactory candidate to be defined as an agglomerating support for other analytes.

Two linear regressions with slightly different gradients were observed in the standardization process with Calcium titrisol and Calcium suppressors. Therefore, they were separated into two groups: the first in the range between 0 and 20 ppm and the second between 20 and 100 ppm. Their results and adjustments are presented in Table 8.

**Table 8:** Calibration curve for standard Ca in 10% HCl with suppressors (*) in NaNO_3_ matrix.

	ppm	Abs	
1	0	0.0015	
2	5	0.0363	Without suppressors
3	10	0.0751	Abs = 0.00701 C + 0.00258
4	15	0.1110	*r* = 0.996
5	20	0.1393	

1	0	0.0006	With suppressors
2	20 (*)	0.0279	Abs = 0.0133 C + 0.0028
3	50 (*)	0.0760	*r* = 0.993
4	75 (*)	0.1020	
5	100 (*)	0.1326	

Between the two adjusted curves, a decision was made to use the first one because it evidenced a slightly better Pearson coefficient than the second one. Accordingly, the instrumentally measured Abs values of the digested pellets following XRF were interpolated from this equation and represented as Ca read as opposed to the gravimetrically observed masses. Their results are presented in Table 9.

**Table 9:** Calcium mass observed according to gravimetry, calculation of read mass according to AAS and final percentage composition of read Ca % (w/w).

	Ca, mg obs	Total pellet mass mg	% Ca mg/mg (w/w) obs	Abs	C, ppm	Ca, mg read	% Ca (w/w) read	Proportionality factor obs/read
1	0	62.9	0	0.0011	−0.20	0.04	0.1	0
2	1.9	68.8	2.8	0.0619	8.50	2.15	3.1	0.89
3	4.9	77.7	6.3	0.1517	21.3	5.87	7.6	0.84
4	7.8	83.4	9.4	0.2381	33.6	9.91	11.9	0.79
5	9.4	87.6	10.8	0.2051	28.9	9.24	10.5	1.03
6	12.6	93.9	13.4	0.3668	52.0	19.00	20.2	0.66
7	14.7	99.9	14.8	0.3785	53.6	18.52	18.5	0.80

	Gravimetry	Gravimetry	Gravimetry	AAS	AAS	AAS	AAS	AAS

*V* = 250 ml 1 ppm = 1 mg/L								0.7 ± 0.1

**Fig. 12:**
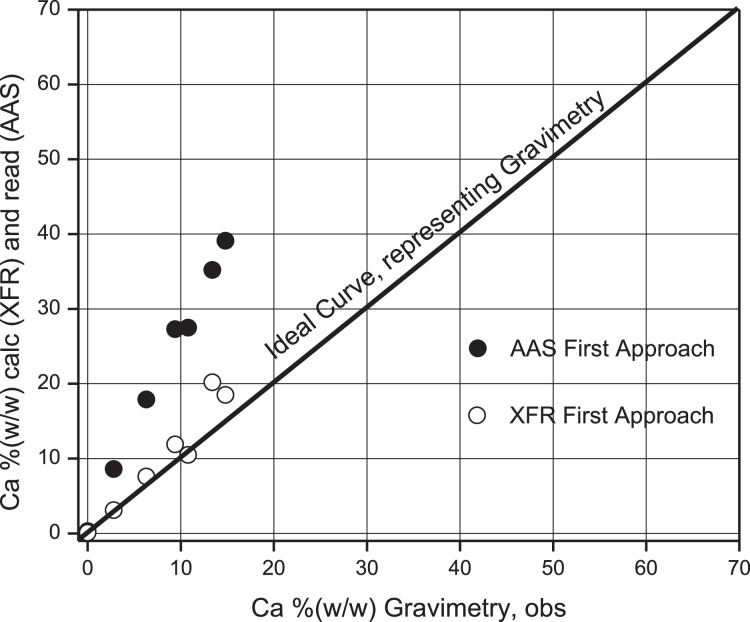
Comparison between% Ca (w/w) obs from Gravimetry and% Ca (w/w) calc from XRF, and from% Ca (w/w) read from AAS, First approach.

As can be seen in this Table, the% (w/w) Calcium percentage read regarding the obs is overestimated by a factor of 0.7 on average with an estimation error of 0.1. An intercomparison between the Gravimetry, AAS, and the values measured using XRF of the ideal samples taken from Table 3 for % Ca calc is presented in Table 10. A correlation between all these values is presented in Fig. 12, considering gravimetry as the ideal curve. A scale of 70–70 was designed to normalize the following discussions in the present work. Table 11

**Table 10:** Intercomparison between the three techniques Gravimetry, XRF Spectroscopic and AAS for first approach.

	% masa Ca obs	% masa Ca calc	% masa Ca read
1	0.0	0.3	0.1
2	2.8	8.6	3.1
3	6.3	17.9	7.6
4	9.4	27.3	11.9
5	10.8	27.5	10.5
6	13.4	35.2	20.2
7	14.8	39.1	18.5

	Gravimetry	XRF	AAS

**Table 11:** Calcium Calibration Curve using 1000 ppm Calcium titrisol and Calcium interferent suppressors.

	ppm	Abs	
1	0	0.0021	
2	5	0.0609	
3	10	0.1138	Abs = 0.01092 + 0.00943 Ca
4	15	0.1516	*r* = 0.999
5	50	0.4808	

**Fig. 13:**
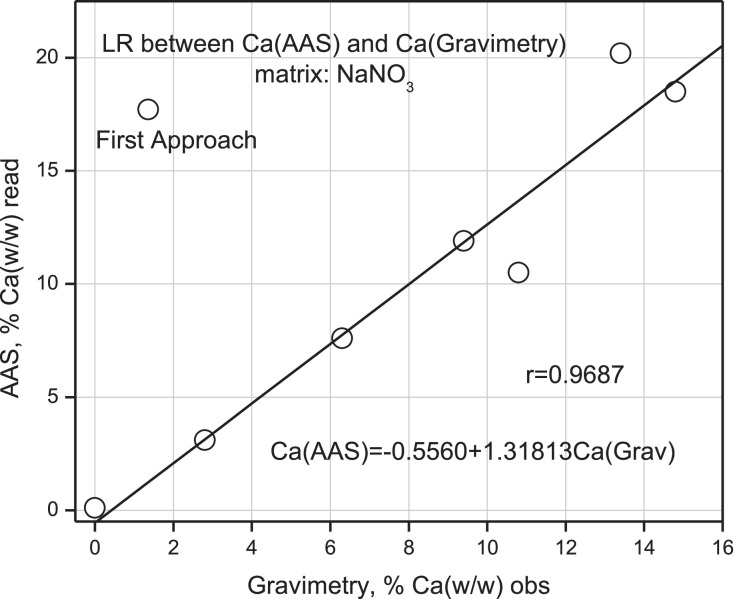
Linear Regression (LR) between% Ca (w/w) read from AAS versus% Ca (w/w) obs from Gravimetry.

**Fig. 14:**
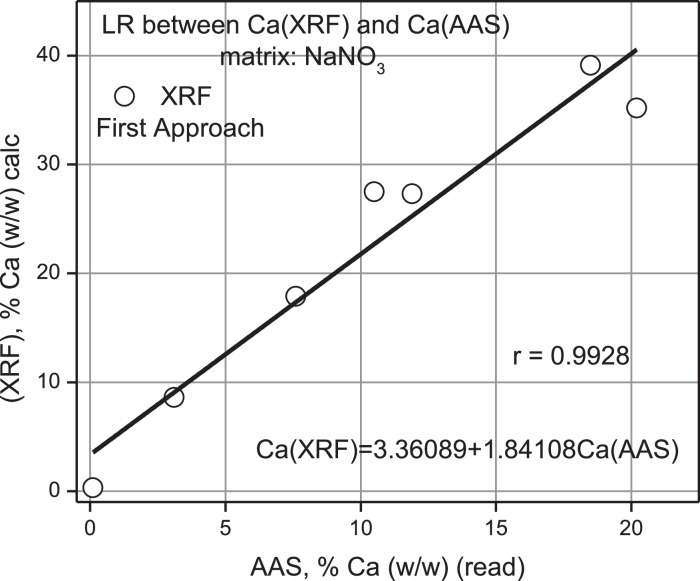
Linear Regression (LR) between% Ca (w/w) calc from XRF versus% Ca (w/w) AAS read.

Calcium % (w/w) values have been taken from this same Table and correlated by simple linear regression between results for % Ca with AAS and gravimetry and % Ca (w/w) between XFR and AAS, respectively. These correlations are presented in Figs. 13 and 14.

As shown in Fig. 13, a simple linear regression between both sets of values produced a Pearson correlation coefficient of 0.9687, which indicates a strong positive correlation, with an overestimated value of 1.3.

The validation of the XRF measurements compared to AAS showed that a linear correlation analysis between the two variables resulted in predicted values overestimated by a systematic error factor of 1.8, indicating inaccuracy in the result but acceptable precision in the fit according to Pearson, *r* = 0.9928, see Fig. 14.

As shown in Fig. 14, a simple linear regression between XRF and AAS values yielded a Pearson correlation coefficient of 0.9928 indicating a strong positive correlation and a 1.8-fold overestimation of XRF values compared to AAS.

##### Second approach

All pellets were digested in HCl/HNO_3_, diluted initially in 50 ml volumetric beakers, and then diluted again to 100 ml. All of them were diluted in 10% HCl.

**Fig. 15:**
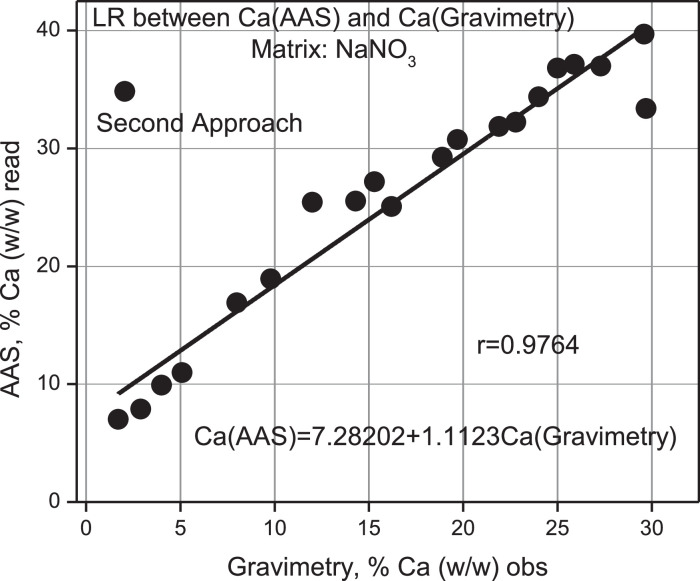
Linear Regression between % Ca (w/w) (AAS) read versus % Ca (w/w) (Grav) obs.

**Fig. 16:**
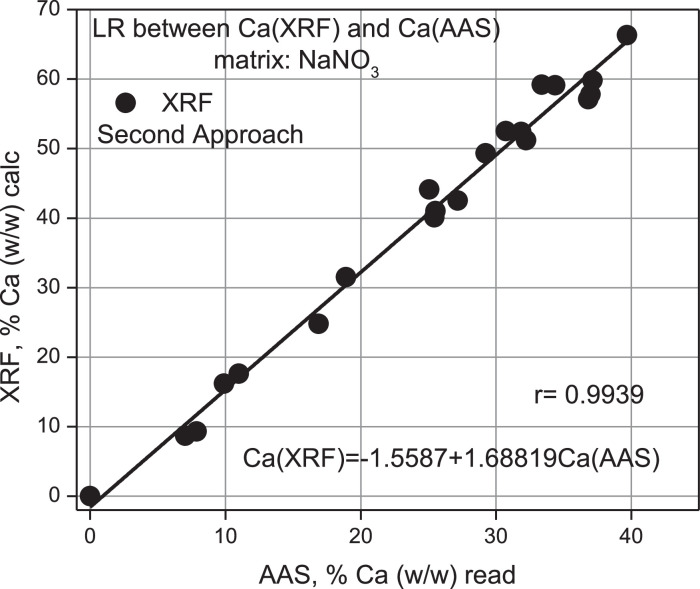
Linear Regression between % Ca (w/w) calc from XRF versus% Ca (w/w) read from AAS.

**Fig. 17:**
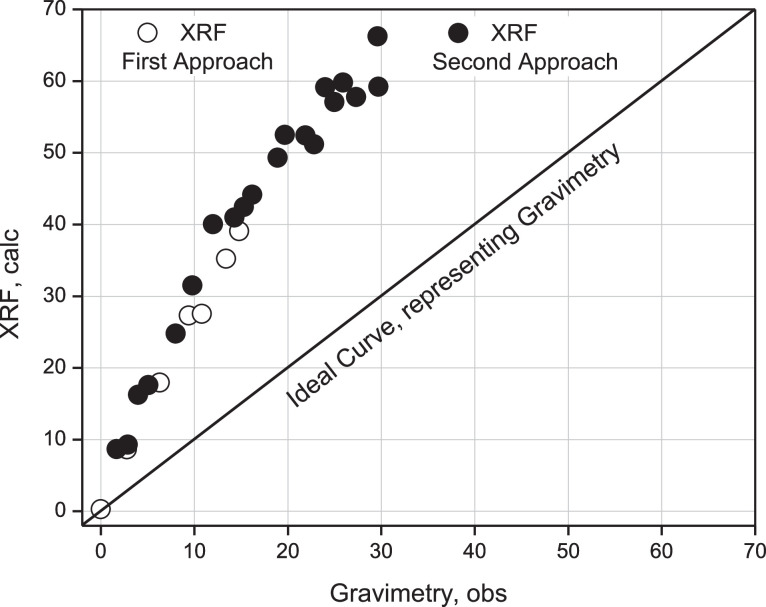
Comparison between ideal curve (Gravimetry) representing obs values, XRF values from first approach and second approach.

**Fig. 18:**
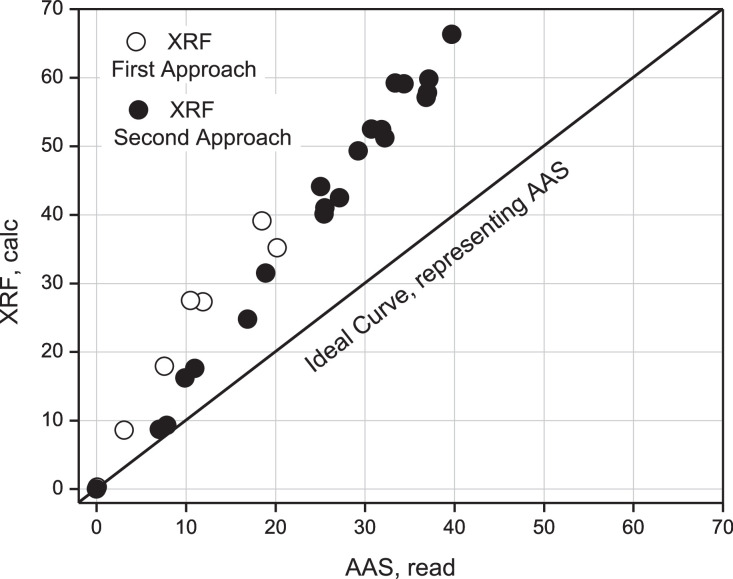
Comparison between ideal curve (AAS) representing read values, XRF calculated values from first and second approach.

According to Table 12, the % (w/w) Calcium percentage read regarding the obs is overestimated by a factor of 0.6 on average with an estimation error of 0.2. An intercomparison between Gravimetry, AAS, and measured values using XRF of the ideal samples taken from Table 5 for % Ca calc is presented in Table 13. From this Table, values have been taken from its columns presented in Figs. 15, 16, 17, and 18.

**Table 12:** Mass of Ca observed according to gravimetry, calculation of mass read according to AAS and final percentage composition of Ca% (w/w). Second Approach.

	Ca, mg obs	Total pellet mass	% Ca (w/w) obs	Abs	C, ppm o mg/L	Ca, mg read	% Ca (w/w) read	Proporcionality Factor obs/read
1	0	0	0	0.0021	−0.9353	0	0	0
2	1.2	69.9	1.7	0.1035	9.8176	4.9088	7.02	0.2
3	2.0	70.2	2.9	0.1151	11.0477	5.5239	7.87	0.4
4	2.8	70.6	4.0	0.1427	13.9745	6.9873	9.90	0.4
5	3.6	70.0	5.1	0.1559	15.3743	7.6872	10.98	0.5
6	5.6	70.4	8.0	0.2352	23.7837	11.8918	16.89	0.5
7	6.8	70.1	9.8	0.2610	26.5196	13.2598	18.92	0.5
8	8.4	70.2	12.0	0.3477	35.7137	17.8568	25.44	0.5
9	10.1	70.3	14.3	0.3494	35.8940	17.9470	25.53	0.6
10	10.8	70.7	15.3	0.3732	38.4178	19.2089	27.17	0.6
11	11.7	72.4	16.2	0.3531	36.2863	18.1432	25.06	0.6
12	13.2	69.9	18.9	0.3964	40.8780	20.4390	29.24	0.6
13	13.8	70.0	19.7	0.4168	43.0414	21.5207	30.74	0.6
14	15.2	69.2	21.9	0.4269	44.1124	22.0562	31.87	0.7
15	15.8	69.4	22.8	0.4327	44.7275	22.3637	32.22	0.7
16	16.9	70.3	24.0	0.4668	48.3436	24.1718	34.38	0.7
17	17.5	70.1	25.0	0.4978	51.6310	25.8155	36.83	0.7
18	18.5	71.7	25.9	0.5131	53.2534	26.6267	37.14	0.7
19	19.2	70.2	27.3	0.5007	51.9385	25.9692	36.99	0.7
20	24.0	80.8	29.7	0.5196	53.9427	26.9714	33.38	0.9
21	20.7	69.8	29.6	0.5334	55.4062	27.7031	39.69	0.7

	gravimetry	gravimetry	gravimetry	AAS	AAS	AAS	AAS	AAS

V_1_ = 50 ml y V_2_ = 100 mL 1 ppm = 1 mg/L								0.6 ± 0.2

**Table 13:** Intercomparison between the three techniques Gravimetry, Spectroscopic XRF and AAS. Second Approach.

	% Ca obs mass	% Ca calc mass	% Ca read mass
1	0	0	0
2	1.7	8.7	7.0
3	2.9	9.3	7.9
4	4.0	16.2	9.9
5	5.1	17.6	10.9
6	8.0	24.8	16.9
7	9.8	31.5	18.9
8	12.0	40.1	25.4
9	14.3	41.0	25.5
10	15.3	42.5	27.2
11	16.2	44.1	25.1
12	18.9	49.3	29.2
13	19.7	52.5	30.7
14	21.9	52.4	31.9
15	22.8	51.2	32.2
16	24.0	59.1	34.4
17	25.0	57.1	36.8
18	25.9	59.8	37.1
19	27.3	57.8	36.9
20	29.7	59.2	33.4
21	29.6	66.3	39.7

	Gravimetry	XRF	AAS

Validation of the AAS measurements compared to Gravimetry, see Fig. 15, shows that a linear correlation analysis between both variables allows arriving at predicted values overestimated by a systematic error factor of 1.11, or equivalent to an inaccuracy of 11% in the result, though, with adequate precision in the adjustment given the strong and positive correlation according to Pearson, *r* = 0.9764.

According to this Figure, it can be seen that the determination of Ca by XRF, in the case of the second approximation, compared to AAS is overestimated by a factor of 1.68, which is equivalent to an error of 68% between both techniques. A slightly better result than the first approximation, see Fig. 14.

A comparative analysis of the results achieved for Calcium determination using the AAS technique between the first and second approximation is presented in Fig. 17. The diagonal line corresponds to the ideal curve provided by gravimetry to measure the observed value of the real value that should be achieved using instrumental validation. It can be seen that both experimental determinations are above the ideal curve, indicating that the tendency to evaluate real samples by AAS is overestimated compared to the alternative curve as a gravimetry-delivered qualifier. This tendency can also be observed among the ideal samples determined by XRF, validated compared to AAS.

Fig. 18 shows an additional comparative analysis between the results achieved for the determination of Calcium using the XRF technique, using the first and second approximation. The diagonal plotted line corresponds to the curve delivered by the AAS technique to measure the read. Therefore, the real value should be achieved using this technique as an instrumental validation in considering the AAS as a standardized technique in literature.

It can be seen that both experimental approaches, according to XRF, are above the AAS curve, now considered a standard. Thus, the tendency to evaluate real samples by XRF is overestimated compared to the alternative curve provided by AAS. It can also be seen that the second approximation is slightly better than the first one.

The validation of ideal samples of the element Calcium using the XRF technique against the gravimetric technique and AAS as experimental standard using linear correlation methods to estimate from the statistical inference that the real concentration of the analyte allowed us to appreciate that both approximations are above the ideal curve, with a value of 1.8 (a relative overestimation error of 80%) against AAS (*r* = 0.9703), to see Fig. 14, and 1.68 (overestimation 68%) against AAS (*r* = 0.9939), to see Fig. 16.

Consequently, the tendency to evaluate calcium samples is overestimated compared to the standard curve, which indicates inaccuracy in the result, although the accuracy of the Pearson's fit is adequate. Since the latter correlation is very close to 1.0 and its statistical inference equation is accurately known, the authors of this work estimate that the quantification protocol could be valid for application to real sediment samples.

Regarding the difference found between the results calculated by XRF and those observed by AAS, a test with similar characteristics to the one reached by this work, oriented to the determination of As, Cu, Pb and Zn, in soils assays was found [5]. In this test, the reported XRF values were slightly biased when compared to that of the AAS analysis, with the slope being lesser than one for Pb and Cu, and slightly more than one for As and Zn analysis. In addition, portable XRF instrument tested in this research are highly correlated with AAS. This has also been verified in the present work, see Fig. 18. Another similar experience was performed by another group [8].The results involving Pb and Zn determination in soil assays agree between both techniques. However, it is not appreciated if the degree of correlation between XRF and AAS shows the same result as in the case of the present work. Other determinations of Pb, Zn, Mn [9] using XRF and AAS shows agreement between both techniques, reaching a 1:1 factor (ED-XRF/AAS) for the elements Ca, Cu, Fe and Zn. In the case of this work, for the Ca element a factor of 1.8:1 (pXRF/AAS) was reached.

On the other hand, according to the literature prospected regarding the determinations of lead in different soil samples were carried out by X-ray fluorescence (XRF) (borate-glass fusion 1∶3), atomic absorption spectrometry (AAS), and induced coupled plasma atomic emission spectrometry (ICP-AES) (aqua regia processes). The concentration ranges in contaminated soils were 10 to 200 wt. ppm and 0.05 to 3.0 wt.%. The detection limit of XRF (5 ppm) is adequate; the precision of AAS (1%) is the best, but the relative accuracy of all three methods does not surpass 10%. The authors pointed out that this was because of sampling and sample preparation errors, the latter being difficult to judge. Determinations with different methods and correlation analysis of the results are necessary [10] .

Finally, the contents of Cr, Ni, Cu, Zn, As, Pb, and Cd in standard soil and two soil samples were determined by XRF and ICP-MS, respectively. The two methods relative error, precision, and accuracy were compared and studied. The contents of Cr, Ni, Cu, Zn, As, Pb, and Cd in standard soil determined by XRF and ICP-MS, respectively, showed that the precision of the two methods could meet the determination standard's requirements. The relative error of XRF in determining Ni, As, Pb, and Cd was more significant than that of ICP-MS. There was no significant difference in the precision of the two methods except for Cd [11].

## Concluding remarks

In this method, an efficient methodology for quantifying the analyte Calcium in soil samples was proposed to allow a representative environmental evaluation. Using a portable XRF instrument, a study of the sample preparation methodology to determine calcium in two ideal matrices was proposed to approach this problem. At the same time, the use of the emission lines of an international Calcium standard, specifically through the use of the area under the curve, as an appropriate observable, allowed to achieve a quantitative and reasonable determination of ideal samples prepared in the System Modeling Laboratory, from Universidad de Tarapacá.

Two sample support matrices were used for XRF: glucose and NaNO_3_. Both exhibited good behavior from the point of view of the absence of emission; however, only the latter allows adequate calcination. The NaNO_3_ matrix is very soluble in an acidic environment; therefore, it is an excellent candidate to be defined as binding support for other analytes.

There were two approaches. The first approach had 7 experimental points, and the second approach had 20 experimental points. The second contained a larger population of data than the first, and therefore its results should have more considerable statistical significance.

Of the two approaches used with the NaNO_3_ matrix, the second one (*N* = 20 points) allows reaching a recommendable linear regression. Consequently, a method for analyzing environmental samples was proposed. This methodology is the contribution of this working group.

Finally, XRF does not require the digestion of samples to determine heavy soil metals, and the instrument is simple to operate and resembles high efficiency. However, the accuracy and comparison with other experimental methods need further study.

## Ethics statements

No statements.

## CRediT author statement

**Claudio H. González-Rojas**: Conceptualization, Methodology, Resources, Data Curation, Writing-Reviewing and Editing **Cristián Castro-Rodriguez**: Visualization, Supervision, Editing **Sebastián Gutiérrez-Vivanco**: Conceptualization, Resources, Validity tests, Data Curation **Esteban Vargas-Vera**: Validation.

## Declaration of Competing Interest

The authors declare that they have no known competing financial interests or personal relationships that could have appeared to influence the work reported in this paper.

## Data Availability

Data will be made available on request.

## Appendix Supplementary materials

Image, application 1

## References

[1] Beckhoff B., Kanngießer B., Langhoff N., Wedell R., Wolff H. (2007).

[2] Jenkins R., De Vries J.L (1973).

[3] Ramsey M.H., Potts P.J., Webb P.C., Watkins P., Watson J.S., Coles B.J. An objective assessment of analytical method precision: comparison of ICP-AES and XRF for the analysis of silicate rocks. 1995.

[4] Jenkins R. (1995).

[5] Radu T., Diamond D. (2009). Comparison of soil pollution concentrations determined using AAS and portable XRF techniques. J. Hazard. Mater..

[6] Kimbrough D.E., Wakakuwa J.R. (1989). Acid digestion for sediments, sludges, soils, and solid wastes. A proposed alternative to EPA SW 846 Method 3050. Environ. Sci. Technol..

[7] Löwemark L., Chen H.F., Yang T.N., Kylander M., Yu E.F., Hsu Y.W. (2011). Normalizing XRF-scanner data: a cautionary note on the interpretation of high-resolution records from organic-rich lakes. J. Asian Earth Sci..

[8] Ene A., Stihi C., Popescu I., Gheboianu A., Bosneaga A., Bancuta I. (2009). Comparative studies on heavy metal content of soils using AAS and EDXRF atomic spectrometric techniques. Ann Dunarea Jos Univ. Galati, Fasc II..

[9] Dulama I.D., Chelarescu E.D., Duliu O. (2016). Heavy metal contents of Brassica oleracea as bioindicator determined by XRF and AAS analytical methods. Rom. Rep. Phys..

[10] Freiburg C., Molepo J.M., Sansoni B. (1987). Comparative determinations of lead in soils by X-ray fluorescence, atomic absorption spectrometry, and atomic emission spectrometry. Fresenius' Zeitsch. Analyt. Chem..

[11] Li W., Niu N., Guo N., Zhou H., Bu J., Ding A. (2021). Comparative Study on the Determination of heavy metals in Soil by XRF and ICP-MS. J. Phys.: Conf. Ser..

