# Supplementary material for: Calcium element quantification model using a portable X-ray fluorescence unit
Source: MethodsX. 2023 Jul 12;11:102287. doi: 10.1016/j.mex.2023.102287 (PMC10372925; doi:10.1016/j.mex.2023.102287)
Supplement: Supplementary file 1 [file mmc1.docx]

**Supplementary material *and/or* additional information**

- **Additional information**

X-ray fluorescence (XRF) is an analytical technique that has been gaining momentum over the last two decades and is used to estimate the composition of rocks, soils and Paleolithic lake sediments. The determination of elements and their variations in lake sediments, for example, provides access to information concerning environmental effects and climatic changes and also the possible anthropogenic influence on lake sediments, marine sediments and alluvial soils. The technique has also provided expertise in the composition of clays and pigments used in objects of archaeological value and lately in medicinal plants.

The advent of portable systems has made the applications of this technique increasingly versatile, and. Depending on the ionizing radiation generator tube used, it can detect from Al to U. Consequently, this technique is fast (less than 5 minutes) and non-destructive, which allows delivering elemental composition information directly from the samples under study. Other techniques applied to determine heavy elements in aerosols are neutron activation analysis (NAA), PIXE, and others.

This technique's usefulness is limited by a few factors, however. The most obvious is that elemental variations are measured as counts rather than concentration and that updating to concentration requires quantitative chemical analysis of the sample bulk. Other factors that influence the measurement of XRF spectra are the water content of the sample, the surface roughness of the sample, grain size variations, chemical interactions in the matrix containing the analytes that lead to scattering or emission subtraction and instrument sensitivity.

The main advantage of this technique over conventional geochemical analysis is that element intensities are obtained directly at the surface of the sediment or target sample. The spatial resolution of the portable devices has a much higher spatial resolution than conventional destructive methods. Nevertheless, the conversion from intensity units to concentration units has not entirely been solved.

The study of soils and sediment material dispersed in the air established Calcium and Iron as the predominant elements in previous tests in the Region of Arica and Parinacota-Chile. Calcium seemed suitable for conducting a study to validate the present technique.

The techniques of analysis validated to date are distinctly ICP-plasma and AAS. In the case of these two techniques, the sample collection and subsequent conditioning and processing llow quantitative determinations to be made with high precision and well-recognized accuracy. In other words, their determination requires a protocol that involves a certain amount of time between sample collection and their measurement in laboratories. In-situ determination is challenging from the point of view of simultaneity of analysis and diagnosis. AAS is one of the most recognized validated techniques in literature for determining heavy elements in soils, water, and aerosolized materials. Another widely used technique is portable X-Ray Fluorescence. Nowadays, the available instrumentation allows the determination of various metals in soil matrices in this analysis modality. The XRF implemented in portable instrumentation is a case in point. This technique, however, provides results in terms of percentage composition in the case of soil analysis. Its results are a function of the soil composition, and the soil is highly diverse. Thus, it does not allow achieving a practical methodology for quantifying an analyte, particularly Calcium, in ppm or similar units that would provide a representative environmental assessment. Two objectives are proposed in this work to address this problem.

The first one is to study the sample preparation methodology for estimating Calcium in two ideal matrices using the portable XRF instrument, examining the instrumental conditions of the portable XRF emitter to define an appropriate observable supporting the quantitative determination of the concentration of the Calcium element. The second objective is to study the amount attained in the emission beads, simultaneously as the analysis of the area under the curve of the Calcium emission line, in % w/w, establishing a means to measure and compare soil samples aerosolized material in quantitative terms. These two techniques have been proposed to validate the measurements made with XRF to achieve these objectives. They are gravimetry and AAS. The first one proposes to use a battery of ideal pellets and examine them by gravimetry. The second one proposes contrasting the same tablets once they have been in the liquid phase employing a calibration curve using flame Atomic Absorption Spectroscopy for the Calcium element.

From the physical background of the XRF measuring principle, it is known that in addition to matrix and grain size effects, the water content has a significant influence on the results of XRF analysis. In this context, a work found that some elements like as (As, Cu, Fe, Pb, K, Ti, Zn) can have deviations of up to 30 % related to the real value. This study proposed work with dehydrated compounds, to minimize the possible real effect on our determination of idealized material.

A work evaluated the precision of predictions for the retention of the preservative chromium and copper arsenate in wood using X-ray fluorescence and near-infrared diffuse reflectance. The quality of the predictions were compared with each other and with retention values obtained experimentally by gravimetric method, atomic absorption, and total ash content. The coefficient of determination between the K-Alpha fluorescence intensities and near-infrared diffuse reflectance regarding conventional methods was between 0,909 and 0,986. The results can be considered acceptable for predictive purposes.
